# An interrupted time-series analysis assessing the association of the COVID-19 pandemic on healthcare-associated infections and antimicrobial-resistant organisms in Canadian acute care hospitals, 2018–2022

**DOI:** 10.1017/ice.2025.10247

**Published:** 2025-10

**Authors:** Anada Silva, Jessica J. Bartoszko, Joëlle Cayen, Kelly B. Choi, Robyn Mitchell, Jeannette L. Comeau, Charles Frenette, Susy S. Hota, Jennie Johnstone, Kevin C. Katz, Stephanie W. Smith, Jocelyn A. Srigley, Kathryn N. Suh, Nisha Thampi

**Affiliations:** 1 Centre for Communicable Diseases and Infection Control (CCDIC), Public Health Agency of Canada, Ottawa, Canada; 2 Dalhousie University, Halifax, Canada; 3 McGill University Health Centre, Montréal, Canada; 4 University Health Network, Toronto, Canada; 5 Sinai Health, Toronto, Canada; 6 North York General Hospital, Toronto, Canada; 7 University of Alberta, Edmonton, Canada; 8 BC Children’s & Women’s Hospitals, Vancouver, Canada; 9 The Ottawa Hospital, Ottawa, Canada; 10 Children’s Hospital of Eastern Ontario, Ottawa, Canada

## Introduction

Canadian acute care hospitals continue to be burdened by healthcare-associated infections (HAIs), including those caused by antimicrobial-resistant organisms (AROs), resulting in increased morbidity, mortality, and excess healthcare costs.^1^ The coronavirus disease 2019 (COVID-19) pandemic caused by the SARS-CoV-2 virus caused major operational changes in hospitals, disrupting infection prevention and control (IPC) and antimicrobial stewardship programs.^2^ Conversely, reduced population mobility and international travel along with greater IPC awareness may have decreased HAI transmission.^2,3^ International studies reported varying changes in HAI and ARO rates during the pandemic.^3^

In Canada, hospital-based surveillance on adult and pediatric HAIs, including AROs, are conducted through the Canadian Nosocomial Infection Surveillance Program (CNISP), a collaboration between the Public Health Agency of Canada, including the National Microbiology Laboratory, the Association of Medical Microbiology and Infectious Disease Canada, and participating sentinel hospitals.

This study assessed the immediate and long-term association between the COVID-19 pandemic and the incidence of healthcare-associated (HA) rates of *Clostridioides difficile* infection (CDI), methicillin-resistant *Staphylococcus aureus* (MRSA) bloodstream infection (BSI), vancomycin-resistant *Enterococcus* (VRE) BSI, carbapenemase-producing *Enterobacterales* (CPE) infections among hospital inpatients, and central line-associated bloodstream infection (CLABSI) in the adult intensive care unit (ICU) in Canadian acute care hospitals participating in CNISP between 2018 and 2022.

## Methods

### Data sources

The study surveillance period spanned 60 months (January 1, 2018–December 31, 2022). We selected five priority HAIs of interest with significant hospital burden: HA-CDI, HA-MRSA BSI, HA-VRE BSI, and HA-CPE infections among all hospital inpatients, and CLABSIs in adult ICUs with a mix of patient types (i.e., medical/surgical). Hospitals may select surveillance participation based on resource capacity and relevance, and thus varied by HAI. For each HAI, we restricted our study sample to hospitals that submitted comprehensive data for all five surveillance years.

Data collection, HAI case definitions, and laboratory methods were previously described.^4^ Monthly patient days were not available and were estimated by dividing the quarterly totals by the number of days in the quarter, then multiplying by the number of days in the month—assuming consistent patient days throughout each quarter and month. Monthly infection rates were calculated per 10,000 patient days by dividing the number of monthly incident infections by monthly patient days, then multiplying by 10,000. For CLABSIs, we calculated monthly infection rates per 1,000 central line days.

### Statistical analysis

We used a generalized linear model with quasi-Poisson distribution, offset by the log number of patient days to perform interrupted time-series modeling to assess changes in monthly infection rates between the pre-COVID-19 pandemic period (January 1, 2018–February 29, 2020; 26 time points) and the COVID-19 pandemic period (March 1, 2020–December 31, 2022; 34 time points). We selected these periods based on the most available and complete hospital infection data to reasonably assess changes in HAI trends.

We adjusted models for seasonality and select hospital-level covariates: geographic region, bed size category, teaching status, and hospital type. We assessed both immediate (step) and long-term (slope) changes using step-and-slope modeling for each eligible HAI. We reported results as incidence rate ratios (IRRs) with 95% confidence intervals (CIs) adjusted for hospital-level clustering. For each infection, a forward addition stepwise procedure was used to select the most parsimonious model. We assessed autocorrelation using plots of the autocorrelation and partial autocorrelation functions. We conducted all analyses using R 4.1.1 at an *α* ≤ 0.05.

## Results

We included a total of 11,267 HAIs in this study from 26–56 Canadian acute care hospitals from 2018 to 2022 (Table 1), with 43% (*n* = 4,862) and 57% (*n* = 6,405) reported during the pre-pandemic and pandemic periods, respectively. Most infections were HA-CDI (*n* = 7,949, 70.5%), followed by HA-MRSA BSI (*n* = 1,277, 11.3%), HA-VRE BSI (*n* = 1,062, 9.4%), adult mixed ICU CLABSIs (*n* = 814, 7.2%), and HA-CPE infections (*n* = 165, 1.5%). The largest number of participating hospitals were from Central Canada (37–50%), medium-sized (42–54%), and teaching hospitals (82–88%). Supplemental information regarding denominators, patient characteristics, and outcomes are available in Tables S2 and S3.

**Table 1.** Characteristics of hospitals participating in the surveillance of healthcare-associated infections, Canadian Nosocomial Infection Surveillance Program, 2018–2022

Infection (No. of participating hospitals)	HA-MRSA BSI (*N* = 44)		HA-VRE BSI (*N* = 56)		HA-CDI (*N* = 38)		HA-CPE infections (*N* = 45)		Adult mixed patient ICU CLABSI (*N* = 26)	
	*n*	(%)	*n*	(%)	*n*	(%)	*n*	(%)	*n*	(%)
Region										
East	7	(16)	15	(27)	6	(16)	7	(16)	4	(15)
West	19	(43)	17	(30)	18	(47)	19	(42)	9	(35)
Central	18	(41)	24	(43)	14	(37)	19	(42)	13	(50)
Hospital type										
Adult	23	(52)	29	(52)	19	(50)	24	(53)	20	(77)
Mixed^*^	13	(30)	16	(29)	13	(34)	13	(29)	6	(23)
Pediatric	8	(18)	11	(20)	6	(16)	8	(18)	N/A^**^	–
Bed size category										
Small	10	(23)	18	(32)	10	(26)	10	(22)	2	(8)
Medium	21	(48)	24	(43)	16	(42)	22	(49)	14	(54)
Large	13	(30)	14	(25)	12	(32)	13	(29)	10	(38)
Teaching hospital status										
Yes	37	(84)	44	(79)	31	(82)	38	(84)	23	(88)
No	7	(16)	12	(21)	7	(18)	7	(16)	3	(12)

* Mixed hospitals include both adult and pediatric patient populations.

** Pediatric hospitals do not have adult mixed ICU wards.

Abbreviations: BSI, bloodstream infection; CPE, carbapenemase-producing *Enterobacterales;* CLABSI, central line-associated bloodstream infections; CDI, *Clostridioides difficile* infection; HA, healthcare-associated; ICU, intensive care unit; MRSA, methicillin-resistant *Staphylococcus aureus*; VRE, vancomycin-resistant *Enterococcus*.

Among the HAIs collected across all hospital inpatients, the overall crude incidence rates per 10,000 patient days from 2018 to 2022 were highest for HA-CDI (3.59), followed by HA-MRSA BSI (0.45), HA-VRE BSI (0.29), and HA-CPE (0.06). For adult mixed ICU CLABSIs, the overall crude incidence rate was 1.40 per 1,000 line-days.

Compared to the pre-pandemic period, crude pandemic period rates per 10,000 patient days increased overall for HA-VRE BSI (0.26 to 0.30, *p* = 0.037) and HA-CPE infections (0.04 to 0.07, *p* = 0.002), remained stable for HA-CDI (3.56 to 3.61, *p* = 0.536), and decreased for HA-MRSA BSI from 0.50 to 0.41 infections per 10,000 patient days (*p* = 0.0009). Additionally, adult mixed ICU CLABSI rates increased from 1.25 to 1.51 infections per 1,000 line-days (*p* = 0.009) during the pandemic period.

**Figure 1.**
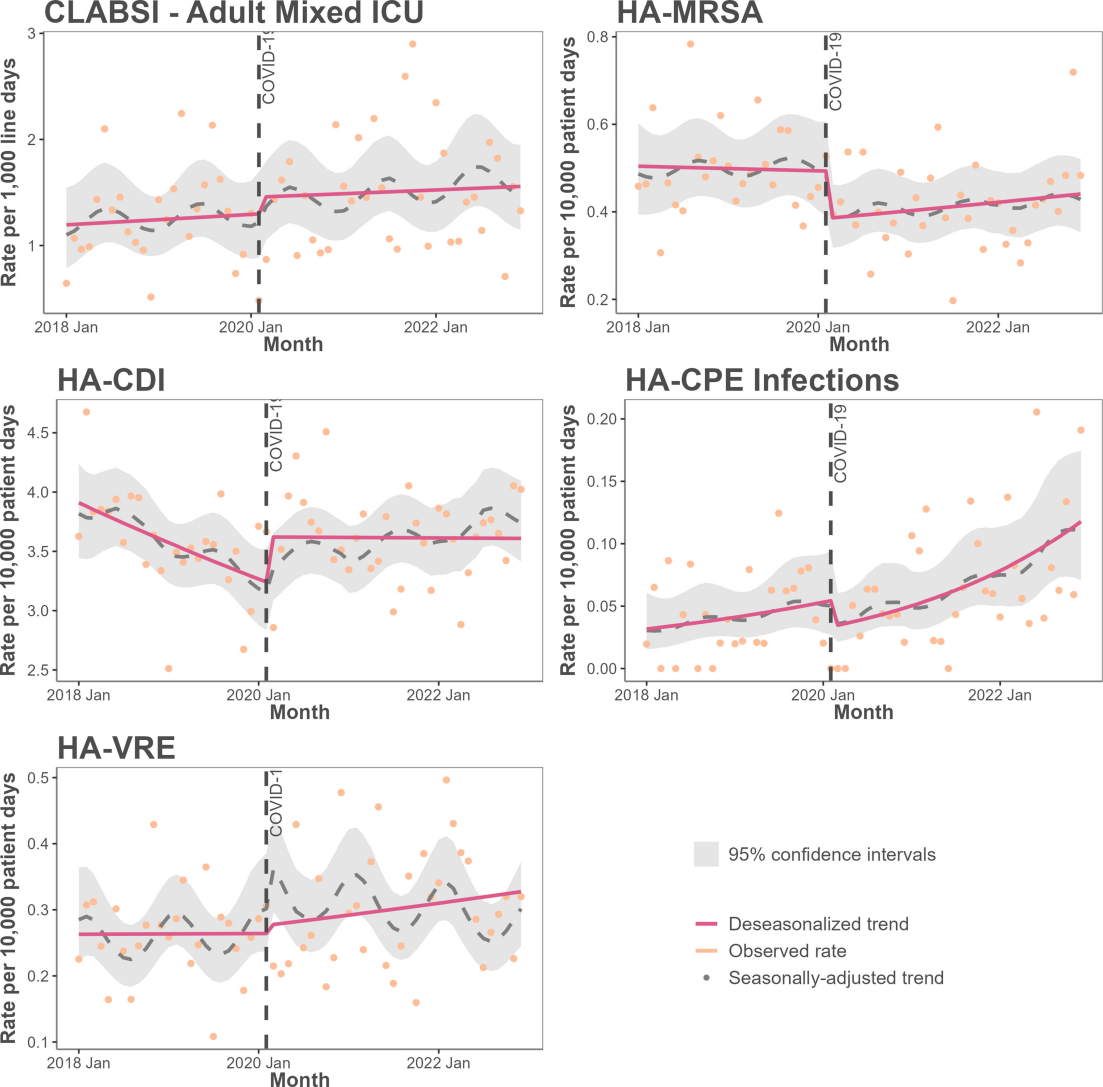
Modeled rates of healthcare-associated infection using interrupted time-series analysis before and during the COVID-19 pandemic, Canadian Nosocomial Infection Surveillance Program, 2018–2022.

Multivariable step-and-slope modeling results (Figure 1, Table S4) indicated that after adjusting for seasonality, clustering, and hospital covariates, the COVID-19 pandemic was significantly associated with a 21% step-change decrease in MRSA BSI rates (aIRR: 0.790 (0.639–0.977), *p* = 0.030) while a significant slope change increase was observed for HA-CDI (aIRR: 1.011 (1.004–1.017), *p* = 0.0007) compared to the pre-pandemic period. During the COVID-19 pandemic, modeled monthly HA-CDI rates remained elevated and stable compared to the declining pre-pandemic trend. We found no significant step or slope changes in monthly rate trends for all other HAIs.

## Discussion

We modeled the association between the COVID-19 pandemic and rates of HAIs including those caused by AROs in a large, national network of Canadian acute care hospitals. We found that the pandemic was associated with a significant immediate step decrease in HA-MRSA BSI rates and a long-term slope increase in HA-CDI rate trends compared to the pre-pandemic period, while all other HAI rate trends did not significantly change.

Our previous surveillance data showed that secular, pre-pandemic rate trends were generally consistent with those observed during the pandemic for all HAIs except HA-CDI.^4^ The pandemic increase in HA-CDI rate trends compared to previously declining rate trends can likely be attributed to competing pandemic-related factors including changes to inpatient acuity, routine care, hospital resources, hospital staffing, and IPC practices.^5^ Monthly pandemic increases in HA-CDI rate trends may be attributed to surges in elderly and vulnerable patient admissions, particularly from Canadian long-term care homes which experienced significant COVID-19 outbreaks.^6^ Additionally, the empiric use of broad-spectrum antibiotics at the start of the pandemic may have disrupted the fecal microbiota in this disproportionately affected elderly population and increased HA-CDI risk.^7^ Pandemic-era HA-CDI rates varied by country, with increases noted in select single-center studies in Europe^7,8^ while decreases were observed in high-income jurisdictions including the US.^3^

Although trends in HA-MRSA BSI rates did not significantly change, step-and-slope modeling resulted in an immediate decrease at pandemic onset. Similar immediate MRSA BSIs decreases were reported in sub-populations in China and South Korea^9,10^ while other studies in France and Brazil observed immediate increases.^2^ The US reported a 12% increase in lab-identified MRSA bacteremia standardized infection ratios at pandemic onset, though attributed to the decline in 2020 Q2 patient days. Increased antimicrobial use including empiric vancomycin at pandemic onset and enhanced IPC practices including hand hygiene, personal protective equipment usage, and environmental cleaning may have offset pandemic-related HAI risk factors and contributed to reductions observed in a Japanese hospital network.^2,11^ System-wide changes in bed assignment policies, visitor restrictions, increased patient and staff vigilance, outpatient care diversion, reduced elective care, and patient-directed healthcare avoidance may have further contributed to these reductions.^12^

Although no significant immediate or long-term changes were observed for HA-VRE BSI, HA-CPE infections, and adult mixed ICU CLABSIs, pre-pandemic increasing trends persisted through the pandemic. Many risk factors such as increased patient acuity, staffing challenges, and device use may have already contributed to rising trends prior to the pandemic.^13^ These risks partially offset by enhanced IPC practices may have resulted in sustained, rather than amplified, increases in these HAIs during the pandemic.

This large, multicenter study included five years of standardized hospital surveillance data for five priority HAIs from all ten Canadian provinces. However, there are limitations to this study. While we captured the first six major COVID-19 pandemic waves, we did not assess the wave-based variation in public health measures, screening practices, antibiotic usage, or dominant SARS-CoV-2 virus strain in our model. Modeled associations were assessed nationally and may differ by hospital due to hospital-specific IPC measures, hospital or community outbreaks, and regional public health measures before and during the pandemic. Future studies should aim to incorporate site-specific COVID-19 hospitalization and ICU admission data to assess the impact of various waves on the burden of HAIs in Canadian acute care settings. Estimating monthly patient and central line days may have not fully captured the monthly variation in patient volume, potentially biasing modeled point estimates and estimates of variance. However, hospital administrative data used as a proxy showed monthly inpatient occupancy variation from March 2020 to June 2021 was under 20%.^12^

## Conclusion

While the COVID-19 pandemic placed a significant burden on the Canadian acute care hospitals, HA-MRSA BSI significantly decreased at its onset, while only HA-CDI rate trends increased compared to the pre-pandemic period. Understanding these pandemic epidemiological effects in the context of changing patient populations and clinical and IPC practices is essential for continued awareness, preparedness, and management of HAIs including those caused by AROs in Canadian acute care settings.

## Supporting information

Silva et al. supplementary material Silva et al. supplementary material

## Supplementary material

To view supplementary material for this article, please visit https://doi.org/10.1017/ice.2025.10247
