# Supplementary material for: An interrupted time-series analysis assessing the association of the COVID-19 pandemic on healthcare-associated infections and antimicrobial-resistant organisms in Canadian acute care hospitals, 2018–2022
Source: Infect Control Hosp Epidemiol. 2025 Sep 10;46(10):1043–7. doi: 10.1017/ice.2025.10247 (PMC12615126; doi:10.1017/ice.2025.10247)
Supplement: Silva et al. supplementary material [file S0899823X2510247Xsup001.docx]

**Table S1.** Monthly median patient admissions, patient days, and central line days among participating hospitals stratified by healthcare-associated infection in the pre-COVID-19 and COVID-19 pandemic periods, Canadian Nosocomial Infection Surveillance Program, 2018–2022.

| Infection | Pre-pandemic period  (Jan 2018–Feb 2020) | | Pandemic period (Mar 2020–Dec 2022) | | *p* |
| --- | --- | --- | --- | --- | --- |
|  | Median | (IQR) | Median | (IQR) |  |
| HA-CDI | | | | | |
| Monthly patient admissions | 1,286 | (517–1,863) | 1,206 | (463–1,723) | **0.030** |
| Monthly patient days | 9,108 | (4,127–14,229) | 9,094 | (4,283–13,911) | 0.6 |
| HA-MRSA BSI | | | | | |
| Monthly patient admissions | 1,433 | (734–2,073) | 1,388 | (642–1,968) | **0.012** |
| Monthly patient days | 10,184 | (5,466–14,763) | 9,747 | (5,356–14,305) | 0.2 |
| HA-VRE BSI | | | | | |
| Monthly patient admissions | 1,268 | (548–1,967) | 1,212 | (506–1,882) | **0.018** |
| Monthly patient days | 9,306 | (4,196–13,973) | 8,946 | (4,274–13,720) | 0.16 |
| HA-CPE infections | | | | | |
| Monthly patient admissions | 1,408 | (728–2,064) | 1,363 | (647–1,935) | **0.012** |
| Monthly patient days | 9,919 | (5,578–14,604) | 9,492 | (5,404–14,149) | 0.2 |
| CLABSI (Adult mixed patient ICU) | | | | | |
| Monthly central line days | 316 | (140–504) | 342 | (195–563) | **<0.001** |
| Monthly patient days | 498 | (266–684) | 498 | (279–694) | 0.4 |

Note: Calculations for monthly median patient admissions, patient days, and patient central line days are based on the total number of hospitals participating in surveillance of each HAI. Individual hospital participation in the surveillance of each HAI may vary based on hospital resource capacity and relevance.
Abbreviations: BSI, bloodstream infection; CPE, carbapenemase-producing *Enterobacterales;* CLABSI, central line-associated bloodstream infections; CDI, *Clostridioides difficile* infection; HA, healthcare-associated; ICU, intensive care unit; IQR, interquartile range; MRSA, methicillin-resistant *Staphylococcus aureus*; VRE, vancomycin-resistant *Enterococcus*.

**Table S2.** Patient characteristics and outcomes stratified by healthcare-associated infection in the pre-pandemic and pandemic periods, Canadian Nosocomial Infection Surveillance Program, 2018-2022.

| Infection | Overall (N=11,267) | Pre-pandemic period (Jan 2018–Feb 2020)  (n=4,862) | Pandemic period  (Mar 2020–Dec 2022)  (n=6,405) | *p* |
| --- | --- | --- | --- | --- |
| HA-CDI^†^ | **n=7,949** | **n=3,446** | **n=4,503** |  |
| Age, years, median (IQR) | 70 (57–81) | 70 (58–81) | 70 (57–81) | 0.91 |
| Sex, female, n/N (%) | 3863/7948 (49%) | 1708/3446 (50%) | 2155/4502 (48%) | 0.13 |
| Days to infection, median (IQR)^*^ | 10 (5–21) | 9 (4–22) | 10 (5–21) | 0.33 |
| Patients in ICU, n/N (%)^**^ | 204/1588 (13%) | 80/689 (12%) | 124/899 (14%) | 0.2 |
| Thirty day all-cause mortality, n/N (%) | 140/1599 (8.8%) | 55/687 (8%) | 85/912 (9.3%) | 0.52 |
| HA-MRSA BSI | **n=1,277** | **n=620** | **n=657** |  |
| Age, years, median (IQR) | 63 (47–76) | 64 (48–77) | 62 (47–74) | 0.1 |
| Sex, female, n/N (%) | 473/1,248 (38%) | 254/620 (41%) | 219/628 (35%) | **0.026** |
| Days to infection, median (IQR)^*^ | 12 (4–33) | 12 (3–34) | 13 (4–32) | 0.66 |
| Patients in ICU, n/N (%)^**^ | 398/1,276 (31%) | 196/619 (32%) | 202/657 (31%) | 0.72 |
| Thirty day all-cause mortality, n/N (%) | 286/1,277 (22%) | 143/620 (23%) | 143/657 (22%) | 0.58 |
| HA-VRE BSI | **n=935** | **n=380** | **n=555** |  |
| Age, years, median (IQR) | 62 (50–70) | 60 (48–69) | 62 (51–71) | 0.06 |
| Sex, female, n/N (%) | 367/935 (39%) | 142/380 (37%) | 225/555 (41%) | 0.33 |
| Days to infection, median (IQR)^*^ | 18 (8–34) | 18 (7–33) | 18 (9–35) | 0.47 |
| Patients in hemodialysis, n/N (%)^¥^ | 322/860 (37%) | 140/343 (41%) | 182/517 (35%) | 0.10 |
| Patients in chemotherapy, n/N (%)^¥^ | 167/860 (19%) | 80/343 (23%) | 87/517 (17%) | **0.018** |
| Patients in ICU, n/N (%)^**^ | 426/860 (50%) | 151/328 (46%) | 275/532 (51%) | 0.11 |
| Thirty day all-cause mortality, n/N (%) | 332/930 (36%) | 128/376 (34%) | 204/554 (37%) | 0.39 |
| HA-CPE infections | **n=165** | **n=53** | **n=112** |  |
| Age, years, median (IQR) | 64 (49–73) | 63 (44–72) | 65 (51–73) | 0.39 |
| Sex, female, n/N (%) | 75/162 (46%) | 27/51 (53%) | 48/111 (43%) | 0.25 |
| Days to infection, median (IQR)^*^ | 19 (9–44) | 22 (13–58) | 16 (8–44) | 0.23 |
| Patients in ICU, n/N (%)^**^ | 20/116 (17%) | 32/40 (20%) | 12/76 (16%) | 0.57 |
| Thirty day all-cause mortality, n/N (%) | 32/154 (21%) | 7/42 (17%) | 25/112 (22%) | **<0.001** |
| CLABSI (Adult mixed patient ICU) | **n=814** | **n=291** | **n=523** |  |
| Age, years, median (IQR) | 62 (50–71) | 62 (50–72) | 61 (49–71) | 0.26 |
| Sex, female, n/N (%) | 278/814 (34%) | 109/291 (37%) | 169/523 (32%) | 0.14 |
| Days from ICU admission to infection, median (IQR) | 11 (6–21) | 13 (6–24) | 11 (6–20) | 0.093 |
| Thirty day all-cause mortality, n/N (%) | 288/811 (36%) | 102/289 (35%) | 186/522 (36%) | 0.89 |

Abbreviations: BSI, bloodstream infection; CPE, carbapenemase-producing *Enterobacterales;* CLABSI, central line-associated bloodstream infections; CDI, *Clostridioides difficile* infection; HA, healthcare-associated; ICU, intensive care unit; IQR, interquartile range; MRSA, methicillin-resistant *Staphylococcus aureus*; VRE, vancomycin-resistant *Enterococcus*.
Note: A full list of patient-level variables collected for each infection are detailed in the Canadian Nosocomial Infection Surveillance Program protocols (https://ipac-canada.org/cnisp-publications).
†Outcome data for CDI (Patients in ICU, thirty-day all-cause mortality) were collected for cases reported in March and April of each year.
¥The proportion of patients undergoing hemodialysis or chemotherapy treatment were only available for HA-VRE BSI surveillance.
*Only cases with healthcare-associated (your acute care facility) acquisition type were included in calculations.
**The proportion of patients in ICU combined those already in ICU and those admitted to ICU within 30 days of first positive culture.

**Table S3.** Infection-specific unadjusted and adjusted incidence rate ratios comparing the pre-pandemic and pandemic periods, Canadian Nosocomial Infection Surveillance Program, 2018-2022.

| Infection | Model component | Unadjusted IRR (95% CI)^†^ | *p* | Adjusted IRR (95% CI)^*^ | *p* |
| --- | --- | --- | --- | --- | --- |
| HA-CDI | Step change | 1.121 (1.001– 1.256) | 0.052 | 1.054 (0.952– 1.168) | 0.312 |
|  | Slope change | 1.008 (1.001–1.014) | **0.028** | 1.011 (1.004– 1.017) | **0.0007** |
| HA-MRSA BSI | Step change | 0.793 (0.623 – 1.010) | 0.066 | 0.790 (0.639– 0.977) | **0.030** |
|  | Slope change | 1.005 (0.991– 1.019) | 0.462 | 1.001 (0.988 – 1.014) | 0.850 |
| HA-VRE BSI | Step change | 1.031 (0.788–1.352) | 0.824 | 1.222 (0.958–1.563) | 0.109 |
|  | Slope change | 1.004 (0.989– 1.020) | 0.582 | 0.993 (0.978 to 1.008) | 0.330 |
| HA-CPE | Step change | 0.638 (0.319–1.282) | 0.203 | 0.702 (0.372–1.341) | 0.276 |
|  | Slope change | 1.016 (0.974–1.059) | 0.461 | 1.010 (0.970 - 1.052) | 0.617 |
| Adult mixed patient ICU CLABSI | Step change | 0.917 (0.675–1.244) | 0.578 | 0.963 (0.712–1.303) | 0.808 |
|  | Slope change | 1.010 (0.990–1.031) | 0.332 | 1.011 (0.990–1.031) | 0.311 |

Abbreviations: BSI, bloodstream infection; CPE, carbapenemase-producing *Enterobacterales;* CLABSI, central line-associated bloodstream infections; CDI, *Clostridioides difficile* infection; HA, healthcare-associated; ICU, intensive care unit; IQR, interquartile range; MRSA, methicillin-resistant *Staphylococcus aureus*; VRE, vancomycin-resistant *Enterococcus*.
†Unadjusted model controlled for seasonality and clustering only.
^*^Adjusted model controlled for seasonality and clustering, and additional hospital-level covariates of interest. All adjusted models controlled for region and bed size category, while HA-VRE BSI additionally controlled for hospital type, and adult mixed patient ICU CLABSI additionally controlled for hospital type and teaching hospital status.
